# Cadmium and selenium blood levels in association with congestive heart failure in diabetic and prediabetic patients: a cross-sectional study from the national health and nutrition examination survey

**DOI:** 10.1186/s13098-024-01556-w

**Published:** 2025-01-09

**Authors:** Renyue Ji, Haisheng Wu, Hongli Lin, Yang Li, Yumeng Shi

**Affiliations:** School of Public Health, LKS Faculty of Medicine, The University of Hong Kong, 7 Sassoon Road, Pok Fu Lam, Hong Kong, SAR China

**Keywords:** Heavy metals, Congestive heart failure, Diabetes, Prediabetes, NHANES

## Abstract

**Background:**

Epidemiological research on the association between heavy metals and congestive heart failure (CHF) in individuals with abnormal glucose metabolism is scarce. The study addresses this research gap by examining the link between exposure to heavy metals and the odds of CHF in a population with dysregulated glucose metabolism.

**Method:**

This cross-sectional study includes 7326 patients with diabetes and prediabetes from the National Health and Nutrition Examination Survey from 2011 to 2018. The exposure variables are five environmental heavy metals—cadmium (Cd), lead (Pb), mercury (Hg), selenium (Se), and manganese (Mn)—and the endpoint is CHF, determined via face-to-face interviews. Logistic regression, weighted quantile sum (WQS), and Bayesian kernel machine learning (BKMR) models were employed to investigate the association between exposure to mixtures of five heavy metals and the odds of having CHF in individuals with diabetes and prediabetes.

**Result:**

Multivariate logistic regression analysis Shows that only blood Cd exhibited a significant linear positive correlation with CHF odds (OR: 1.26, 95%CI 1.07–1.47, p = 0.005), there was a significant 14% decrease in the odds rate of CHF for each additional standard deviation of log10 Se (OR: 0.86,95%CI 0.76–0.96, P = 0.009). The WQS index for the metal mixture only marginally increased the odds of CHF by 1% (OR = 1.01, 95% CI 1.00–1.02, P = 0.032). BKMR analysis demonstrated a positive association between Cd levels and the odds of CHF, an inverse relationship with Se levels in patients with diabetes and prediabetes. However, no significant association was observed between the metal mixture and CHF.

**Conclusion:**

This cross-sectional study demonstrates that increased Cd levels are associated with a higher odds of CHF in patients with diabetes and pre-diabetes, whereas elevated blood Se levels significantly mitigate this odds.

**Supplementary Information:**

The online version contains supplementary material available at 10.1186/s13098-024-01556-w.

## Introduction

Congestive heart failure (CHF), the final manifestation of various cardiovascular diseases, is a complex clinical syndrome characterized by heterogeneous pathophysiological mechanisms and multiple pathogenic factors [1]. CHF impacts a global population of 56 million individuals, imposing significant burdens on both individuals and society [1]. This condition leads to diminished patient quality of life, elevated rates of hospital readmission, substantial medical expenses, and increased mortality rates [1–3]. Among American adults, the odds of CHF ranks second globally [4]. Currently, approximately 6.7 million Americans aged over 20 are affected by CHF, with this number projected to escalate to 8.5 million by 2030 [5]. A comprehensive survey conducted in China in 2017, examining CHF within a sample of 52.4 million urban medical insurance beneficiaries aged 25 years and older across six provinces, revealed an estimated total of 12.1 million individuals with CHF, with a odds rate of 1.1% among the population over 25 years of age. The annual incidence of new CHF cases was determined to be 2.97 million, with an average of 3.3 hospitalizations per year for patients with CHF, and 59.8% of these patients being hospitalized at least twice within the year. The average annual hospitalization cost for patients with CHF was calculated at 29,746 yuan, while the average annual outpatient expense was 6023 yuan [6].

Our foremost proposition is to reduce the substantial burden of CHF on individuals and society. We are acquainted with two distinct categories of risk factors for CHF: lifestyle-related factors and disease-related factors. Lifestyle-related factors include smoking, physical inactivity, alcoholism, and socio-economic disadvantage; whereas disease-related factors comprise hypertension, diabetes, hyperlipidemia, and obesity [7, 8]. The recent Universal Definition and Classification of CHF has identified diabetes mellitus as one of the most relevant risk factors for CHF [9]. It is estimated that 10–30% of patients with diabetes mellitus exhibit clinical manifestations of CHF [10]. The underlying pathological mechanisms may involve the development of cardiac dysfunction and increased risk of CHF due to the cumulative effects of chronic hyperglycemia and resultant oxidative stress on cardiac tissue [11, 12]. Pre-diabetes is associated with an increased risk of CHF and a higher incidence of cardiovascular events in patients with CHF, with risks intermediate between those observed in individuals with diabetes and those with normal glucose tolerance [13–15]. Consequently, there is a compelling need to focus on patients with dysregulated glucose metabolism and to identify more sensitive biomarkers, as these efforts are of significant importance for the prevention and progression of HF.

The impact of our surrounding environment is often overlooked, and environmental heavy metals constitute another significant risk factor for cardiovascular disease and diabetes [16]. Currently, an increasing body of evidence suggests that metals such as lead (Pb), mercury (Hg), and cadmium (Cd) are implicated in the etiology of cardiovascular diseases, thereby significantly elevating the risk of developing such conditions [17–19]. Previous studies have demonstrated the favorable impact of selenium (Se) and manganese (Mn) on cardiovascular disease and mortality [20–23]. These five environmental exposures are widely prevalent in the natural environment [24], and individuals frequently encounter them simultaneously through household wastewater, cosmetics, and dietary intake [25]. Therefore, a comprehensive evaluation of the impact of specific metal elements on cardiovascular diseases is warranted. It is imperative to consider the collective influence of these metal exposures on cardiovascular diseases in order to identify the most influential heavy metal elements for such conditions. This holds significant clinical value in formulating prevention and treatment strategies for cardiovascular diseases. Moreover, given the significance of CHF as a terminal stage in cardiovascular disease and its higher odds among individuals with pre-diabetes and diabetes [26, 27], it is imperative to prioritize this specific cohort.

Hence, to address the aforementioned issues, we investigated the association between exposure to mixtures of heavy metals and the odds of CHF in individuals with diabetes and pre-diabetes. Our study aims to provide a comprehensive understanding of the role of environmental heavy metals in the development of CHF, particularly in the context of dysregulated glucose metabolism, and to contribute to the development of targeted prevention and treatment strategies.

## Methods

### Population

Participants in this study were drawn from the National Health and Nutrition Examination Survey (NHANES), a program conducted between 2011 and 2018. Established in the 1960s, NHANES has been conducted biennially since 1999, focusing on assessing the health and nutritional status of the U.S. civilian population. Its objectives include monitoring trends in major diseases, evaluating the effectiveness of public health interventions, and providing data on nutritional intake and health-related risk factors. The survey comprises a detailed in-person interview and a comprehensive physical examination, which includes laboratory tests. The research protocol has received ethical approval from the Research Ethics Review Committee at the National Center for Health Statistics (NCHS), while all participants provided informed consent prior to their inclusion in the study. The specific study design and methodology have been published elsewhere [28, 29], and for more detailed information about NHANES can be obtained from official website: https://www.cdc.gov/nchs/nhanes/index.htm..

**Fig. 1:**
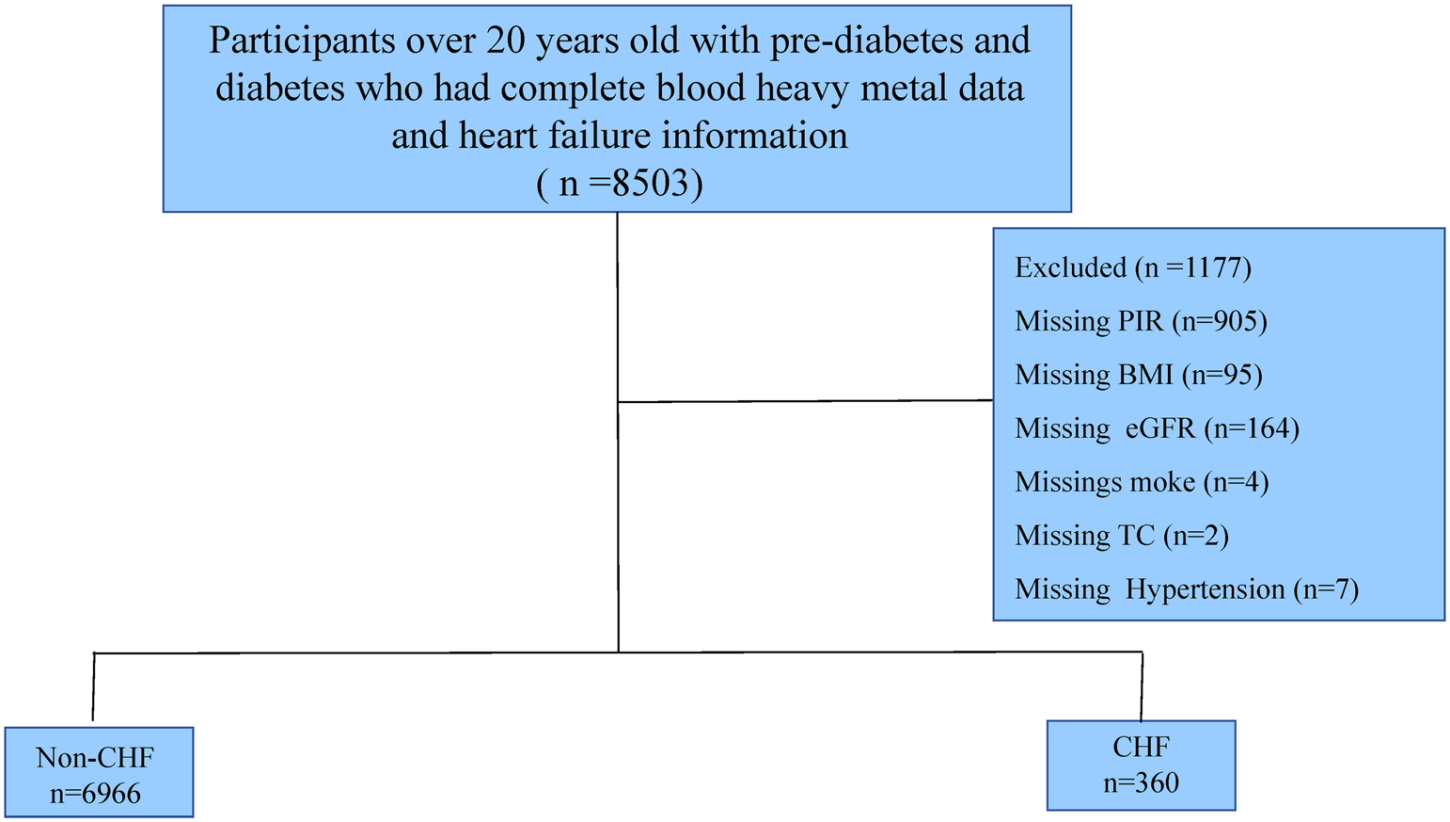
Flowchart of study’participants

In our cross-sectional study, we initially selected 8503 participants over 20 years old with pre-diabetes and diabetes who had complete blood heavy metal data and CHF information. After excluding those missing data on poverty income ratio (PIR, n = 905), body mass index (BMI, n = 95), estimated glomerular filtration rate (eGFR, n = 164), smoking status (n = 4), total cholesterol (TC, n = 2), and hypertension (n = 7), 7326 participants were included in the final analysis. This process is illustrated in Fig. 1.

### Heavy metals measurement

The blood samples were collected from patients who underwent a fasting period exceeding 8 h. Subsequently, the whole blood was preserved at − 30 degrees Celsius and transported to the National Environmental Health Center for subsequent analysis. The concentrations of blood Cd, Pb, Hg, Se, and Mn were quantified using inductively coupled plasma mass spectrometry (ICP-MS). The laboratory has implemented rigorous quality control procedures to ensure the precision and dependability of the measurements. Laboratory details of the methods and quality control data are available at https://wwwn.cdc.gov/Nchs/Nhanes/2011-2012/PBCD_G.htm.

### Congestive heart failure

The assessment of CHF was conducted through face-to-face interviews administered by trained medical professionals. Specifically, participants were asked the question, “Have doctors or other medical professionals ever diagnosed you with CHF?” A positive response to this inquiry indicated the presence of CHF [30]. Furthermore, previous literature has corroborated the reliability of self-reported heart failure as an endpoint [31, 32].

### Covariates

Baseline questionnaires were utilized to collect covariate information, encompassing age, gender, race, smoking status, PIR, BMI, and self-reported baseline medical history including hypertension, hyperlipidemia, and drug use. BMI was determined by measuring height and weight. Blood biochemical markers comprised TC, high-density lipoprotein levels (HDL), and eGFR.The eGFR was calculated using the chronic kidney disease epidemiological cooperation (CKD-EPI) formula [33]. Prediabetes was defined according to the 2013 American Diabetes Association guidelines as meeting any one of the following criteria: HbA1c between 5.7% and 6.4%, fasting plasma glucose (FPG) between 5.6 mmol/L and 6.9 mmol/L, or a 2-h plasma glucose value between 7.8 mmol/L and 11.0 mmol/L during an oral glucose tolerance test (OGTT) [34]. Diabetes was defined as a self-reported physician diagnosis of diabetes or having an HbA1c level ≥ 6.5%, FPG level ≥ 7.0 mmol/L, or a 2-h OGTT plasma glucose level ≥ 11.1 mmol/L.

### Statistical analyses

To account for the non-normal distribution of heavy metals in blood, we applied a logarithmic transformation (log10) to the five heavy metals, thereby approximating their distribution to a normal distribution. The Pearson correlation coefficient is employed to ascertain the correlation between the concentrations of these five heavy metals. The representation of continuous normal variables is typically expressed as the mean (standard deviation), while non-normal distributions are often presented using the median (interquartile ranges) (IQRs). On the other hand, classified variables are commonly depicted in terms of numerical values and corresponding percentages. The chi-square test and t-test or mann–whitney test are employed to assess the association between participants' demographic characteristics and their CHF status.

In order to comprehensively assess the impact of heavy metals on the odds of CHF, we employed three statistical methodologies. Firstly, we conducted multivariate logistic regression analysis to examine the individual effects of each heavy metal on CHF odds and developed three distinct models as described below. The crude model did not adjust for any covariates. Model 1 adjusted for gender, age, race, PIR, and BMI. Additionally, model 2 further adjusted based on model 1 by including TC, HDL-c, eGFR, smoking status, hypertension, hyperlipidemia, lipid-lowering drugs and antihypertensive drugs. Furthermore, the weighted range and WQS regression method was employed to investigate the overall impact of metals on the odds of CHF, as this approach has demonstrated strong performance in characterizing environmental mixtures [35]. Upon observing a significant WQS index, we examined the corresponding weights to ascertain the relative contribution of each heavy metal within the index towards CHF odds. The final outcome is interpreted as the collective effect resulting from an incremental increase in mixed metal concentrations on CHF. Finally, a Bayesian variable selection framework was employed to comprehensively investigate the overall impact of heavy metal mixtures on the odds of CHF. Specifically, the BKMR model compared the influence between exposure levels in specific quartiles and the median. The contribution of each heavy metal to CHF odds was assessed by calculating posterior inclusion probabilities (PIPs), with a significance threshold set at 0.5. Additionally, after adjusting for all covariates, the model utilized a Markov chain Monte Carlo algorithm with 10,000 iterations.

The analyses, including WQS and BKMR, were conducted using R software (version 4.2.3). A significance level of p < 0.05 was considered statistically significant.

## Results

### Population characteristics

**Fig. 2:**
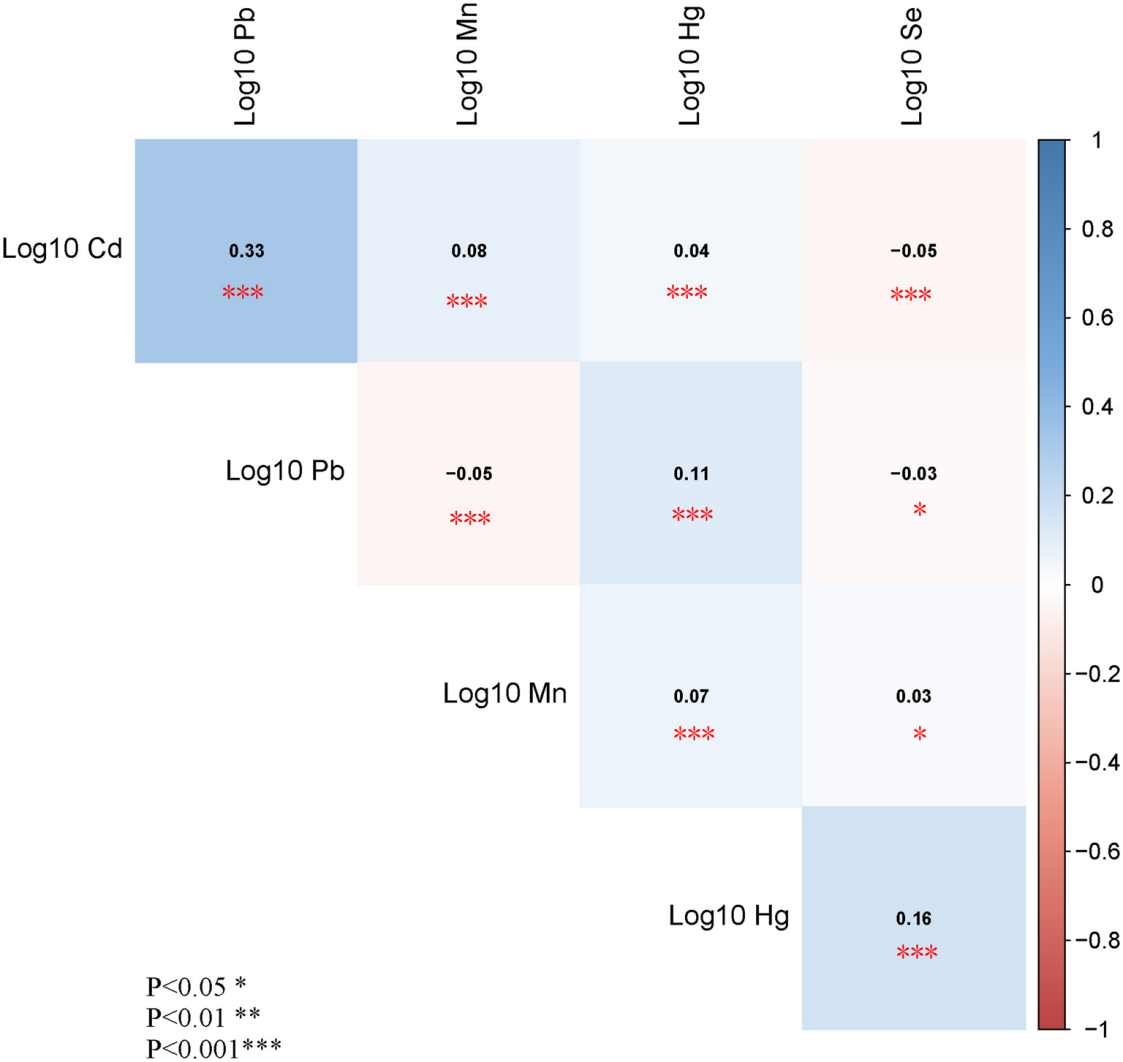
Pearson correlations among the five heavy metals (all P < 0.05). *Pb* lead, *Cd* cadmium, *Hg* mercury, *Se* selenium, *Mn* manganese

A total of 7326 patients diagnosed with prediabetes and diabetes were included in this study, with an average age (SD) of 55.7 (15.8) years. Among them, 51.5% (3772) were male, and chronic congestive heart failure was present in 360 individuals (4.91%), while hypertension affected 3931 individuals (53.7%) and hyperlipidemia affected 5844 individuals (79.8%). The baseline characteristics of demographic and clinical manifestations of participants with or without congestive heart failure are presented in Table 1. Compared to the control group, patients with CHF exhibited older age, a higher proportion of non-hispanic white, and a lower odds of current smokers. Additionally, they had higher values of PIR, BMI, blood Cd and Pb levels, as well as an increased odds of hypertension and hyperlipidemia. Furthermore, the rate of antihypertensive drug usage and lipid-lowering drug intake was higher among CHF patients. Notably, there were significant differences between the CHF group and the control group in terms of low TC, HDL-c, Mn, Hg and Se levels (p < 0.05). However, no gender-based differences were observed between the two groups. The Pearson correlation coefficient between heavy metal concentrations after log10 transformation is presented in Fig. 2. The correlation coefficients between Cd and Pb, Mn, Hg, and Se are 0.33, 0.08, 0.04, and − 0.05, respectively. The coefficients for Pb and Mn, Hg and Se are − 0.05, 0.11, and − 0.03, respectively. For Mn and Hg, and Se, the coefficients are 0.07 and 0.03, respectively, and for Hg and Se, the coefficient is 0.16. All correlation coefficients for heavy metals were statistically significant with *P* values less than 0.05.

**Table 1:** Baseline characteristics of study participants^a^

Variable	Total	Non-CHF	CHF	P value
N	7326	6966	360	
Age,y	55.7 (15.8)	55.0 (15.8)	67.6 (11.4)	< 0.001
Gender, n (%)				
Male	3772 (51.5%)	3572 (51.3%)	200 (55.6%)	0.126
Female	3554 (48.5%)	3394 (48.7%)	160 (44.4%)	
Race				
Non-Hispanic White, N(%)	2588 (35.3%)	2407 (34.6%)	181 (50.3%)	< 0.001
Non-Hispanic Black, N(%)	1795 (24.5%)	1705 (24.5%)	90 (25.0%)	
Mexican American, N(%)	972 (13.3%)	944 (13.6%)	28 (7.78%)	
Other Hispanic, N(%)	768 (10.5%)	737 (10.6%)	31 (8.61%)	
Other races, N(%)	1203 (16.4%)	1173 (16.8%)	30 (8.33%)	
PIR, n (%)	2.45 (1.60)	2.48 (1.61)	1.93 (1.35)	< 0.001
BMI, kg/m^2^	30.9 (7.46)	30.8 (7.38)	33.3 (8.59)	< 0.001
TC, mg/dL	193 (43.1)	194 (42.7)	170 (43.6)	< 0.001
HDL-c, mg/dL	51.3 (15.3)	51.4 (15.3)	47.5 (13.8)	< 0.001
eGFR, mL/min/1.73 m^2^	88.5 (24.0)	89.8 (23.3)	65.0 (24.8)	< 0.001
Smoking status, n (%)				
No	3956 (54.0%)	3805 (54.6%)	151 (41.9%)	< 0.001
Former	2028 (27.7%)	1875 (26.9%)	153 (42.5%)	
Current	1342 (18.3%)	1286 (18.5%)	56 (15.6%)	
Hypertension, n (%)				
No	3395 (46.3%)	3339 (47.9%)	56 (15.6%)	< 0.001
Yes	3931 (53.7%)	3627 (52.1%)	304 (84.4%)	
Hyperlipidemia, n (%)				
No	1482 (20.2%)	1450 (20.8%)	32 (8.89%)	< 0.001
Yes	5844 (79.8%)	5516 (79.2%)	328 (91.1%)	
Lipoprotein-lowering drugs, n (%)				
No	5046 (68.9%)	4927 (70.7%)	119 (33.1%)	< 0.001
Yes	2280 (31.1%)	2039 (29.3%)	241 (66.9%)	
Antihypertensive drugs, n (%)				
No	3983 (54.4%)	3947 (56.7%)	36 (10.0%)	< 0.001
Yes	3343 (45.6%)	3019 (43.3%)	324 (90.0%)	
Cadmium, medium (IQR), μg/L	0.33 [0.20–0.59]	0.33 [0.20–0.58]	0.42 [0.25–0.72]	< 0.001
Lead, medium (IQR), μg/dL	1.11 [0.72;1.71]	1.10 [0.71;1.70]	1.29 [0.90–1.99]	< 0.001
Mercury, medium (IQR), μg/L	0.78 [0.41–1.64]	0.79 [0.42–1.68]	0.63 [0.35–1.19]	< 0.001
Manganese, medium (IQR), μg/L	9.25 [7.40–11.6]	9.27 [7.44–11.7]	8.49 [6.82–10.8]	< 0.001
Selenium, medium (IQR), μg/L	192 [177–208]	192 [178–208]	184 [169–202]	< 0.001

^a^Values are mean (SD), median [IQR] for skewed variables, or n (%) for categorical variables

*CHF* Congestive heart failure, *BMI* body mass index, *PIR* sta. *TC*, total cholesterol, *HDL-C* high-density lipoprotein cholesterol, *eGFR* estimated glomerular filtration rate

### Heavy metals exposure and CHF odds in the logistic regression model

Multivariate logistic regression analysis was conducted to assess the potential association between blood heavy metal concentrations and the odds of CHF. Table 2 presents the single relationships between CHF and each of the five blood heavy metal concentrations. Notably, in fully adjusted model 2, only blood Cd exhibited a significant linear positive correlation with CHF odds (odds ratios (ORs): 1.26, 95% confidence interval [CI]: 1.07–1.47, p = 0.005). When Cd was utilized as a categorical variable, the odds of CHF in the Q4 (Cd ≥ 058) group exhibited a significant 60% increase (95%CI 1.05–2.43, p = 0.028) compared to the participants in the Q1 (Cd < 0.20) group (*P* for trend < 0.05). For each standard deviation (SD) increment in Log Pb levels, the odds of CHF increase by a factor of 1.06 (95% CI 0.93–1.21, p = 0.401), although this increase is not statistically significant. When Pb levels are treated as a categorical variable, the odds of CHF in the Q2 (0.72 < Pb < 1.10), Q3 (1.10 ≤ Pb < 1.70), and Q4 (Pb ≥ 1.70) groups, compared to the Q1 (Pb < 0.72) group, increase by 75%, 62%, and 57%, respectively. Given that the p-value for trend is 0.202, a non-linear correlation between Pb exposure and CHF is suggested. In the categorical analysis of Hg, there was no significance, but in the continuous variable, it was, showing an inverse relationship. Correlation analysis reveals a positive correlation between Se and Hg, which may be attributed to the confounding effect of Se. Thus, after adjusting for Se in the regression analysis, there is no correlation between CHF and Hg (supplementary Table 1). There is no significant association between the odds of CHF and Mn. Furthermore, our findings indicate a linear negative correlation between Se and the odds of CHF, regardless of whether Se is considered as a continuous or classified variable.

**Table 2:** Multivariate logistic regression analysis of heavy metals for the odds of CHF

Exposure	Crude model		Model I		Model II	
	Crude OR (95%CI)	P-value	Adjusted OR (95%CI)	P-value	Adjusted OR (95%CI)	P-value
Log10 Cd (Per SD increment)	1.26 (1.13, 1.39)	< 0.0001	1.26 (1.12, 1.43)	0.0002	1.26 (1.07, 1.47)	0.005
Q1 ( Cd < 0.20)	Reference		Reference		Reference	
Q2 (0.20 < Cd < 0.33)	1.27 (0.89, 1.81)	0.192	1.03 (0.71, 1.49)	0.877	1.06 (0.73, 1.56)	0.745
Q3(0.33 < Cd < 0.58)	2.03 (1.46, 2.82)	< 0.0001	1.43 (1.00, 2.03)	0.048	1.43 (0.99, 2.07)	0.060
Q4 (Cd ≥ 0.58)	2.00 (1.43, 2.78)	< 0.0001	1.69 (1.19, 2.41)	0.004	1.60 (1.05, 2.43)	0.028
P for trend	< 0.0001		0.0004		0.010	
Log10 Pb (Per SD increment)	1.33 (1.20, 1.47)	< 0.0001	1.07 (0.94, 1.22)	0.300	1.06 (0.93, 1.21)	0.401
Q1(Pb < 0.72)	Reference		Reference		Reference	
Q2 (0.72 < Pb < 1.10)	2.51 (1.73, 3.64)	< 0.0001	1.73 (1.17, 2.56)	0.006	1.75 (1.17, 2.62)	0.006
Q3(1.10 < Pb < 1.70)	2.60 (1.79, 3.76)	< 0.0001	1.59 (1.07, 2.36)	0.023	1.62 (1.08, 2.45)	0.021
Q4(≥ 1.70)	2.99 (2.08, 4.31)	< 0.0001	1.53 (1.01, 2.30)	0.043	1.57 (1.03, 2.41)	0.038
P for trend	< 0.0001		0.247		0.202	
Log10 Hg (Per SD increment)	0.76 (0.68, 0.85)	< 0.0001	0.84 (0.74, 0.95)	0.007	0.86 (0.75, 0.98)	0.025
Q1(Hg < 0.41)	Reference		Reference		Reference	
Q2 (0.41 < Hg to < 0.78)	0.94 (0.71, 1.23)	0.642	0.91 (0.68, 1.22)	0.534	0.93 (0.69, 1.26)	0.643
Q3(0.78 < Hg < 1.63)	0.73 (0.54, 0.98)	0.036	0.82 (0.60, 1.12)	0.213	0.85 (0.62, 1.18)	0.339
Q4(Hg ≥ 1.63)	0.53 (0.38, 0.73)	< 0.0001	0.68 (0.48, 0.96)	0.028	0.71 (0.50, 1.01)	0.058
P for trend	< 0.0001		0.024		0.056	
Log10 Mn (Per SD increment)	0.79 (0.71, 0.88)	< 0.0001	0.94 (0.84, 1.06)	0.303	0.99 (0.88, 1.11)	0.838
Q1(Mn < 7.40)	Reference		Reference		Reference	
Q2(7.40 < Mn < 9.25)	0.67 (0.50, 0.89)	0.006	0.79 (0.58, 1.06)	0.118	0.86 (0.63, 1.17)	0.342
Q3(9.25 < Mn < 11.60)	0.71 (0.54, 0.95)	0.019	0.92 (0.69, 1.25)	0.609	1.05 (0.77, 1.43)	0.776
Q4(Mn ≥ 11.60)	0.54 (0.40, 0.73)	< 0.0001	0.85 (0.61, 1.18)	0.329	0.90 (0.64, 1.26)	0.528
P for trend	0.0002		0.470		0.794	
Log10 Se (Per SD increment)	0.73 (0.65, 0.81)	< 0.0001	0.79 (0.71, 0.89)	< 0.0001	0.86 (0.76, 0.96)	0.009
Q1(Se < 177)	Reference		Reference		Reference	
Q2(177 < Se < 192)	0.63 (0.48, 0.83)	0.001	0.70 (0.52, 0.93)	0.015	0.75 (0.56, 1.01)	0.059
Q3(192 < Se < 208)	0.47 (0.34, 0.63)	< 0.0001	0.55 (0.40, 0.75)	0.0002	0.62 (0.45, 0.86)	0.004
Q4(Se ≥ 208)	0.50 (0.37, 0.67)	< 0.0001	0.59 (0.43, 0.81)	0.0009	0.69 (0.50, 0.96)	0.028
P for trend	< 0.0001		0.0001		0.009	

*Pb* lead, *Cd* cadmium, *Hg* mercury, *Se* selenium, *Mn* manganese, *OR* odd ratio, *CI* confidence interval

Non-adjusted model adjust for: None

Adjust I model adjust for: gender, age, race, PIR, BMI

Adjust II model adjust for: gender, age, race, PIR, BMI, TC, HDL-c, eGFR, smoking status, hypertension, hyperlipidemia, lipoprotein-lowering drugs, antihypertensive drugs

### Heavy metals exposure and CHF odds in WQS model

**Fig. 3:**
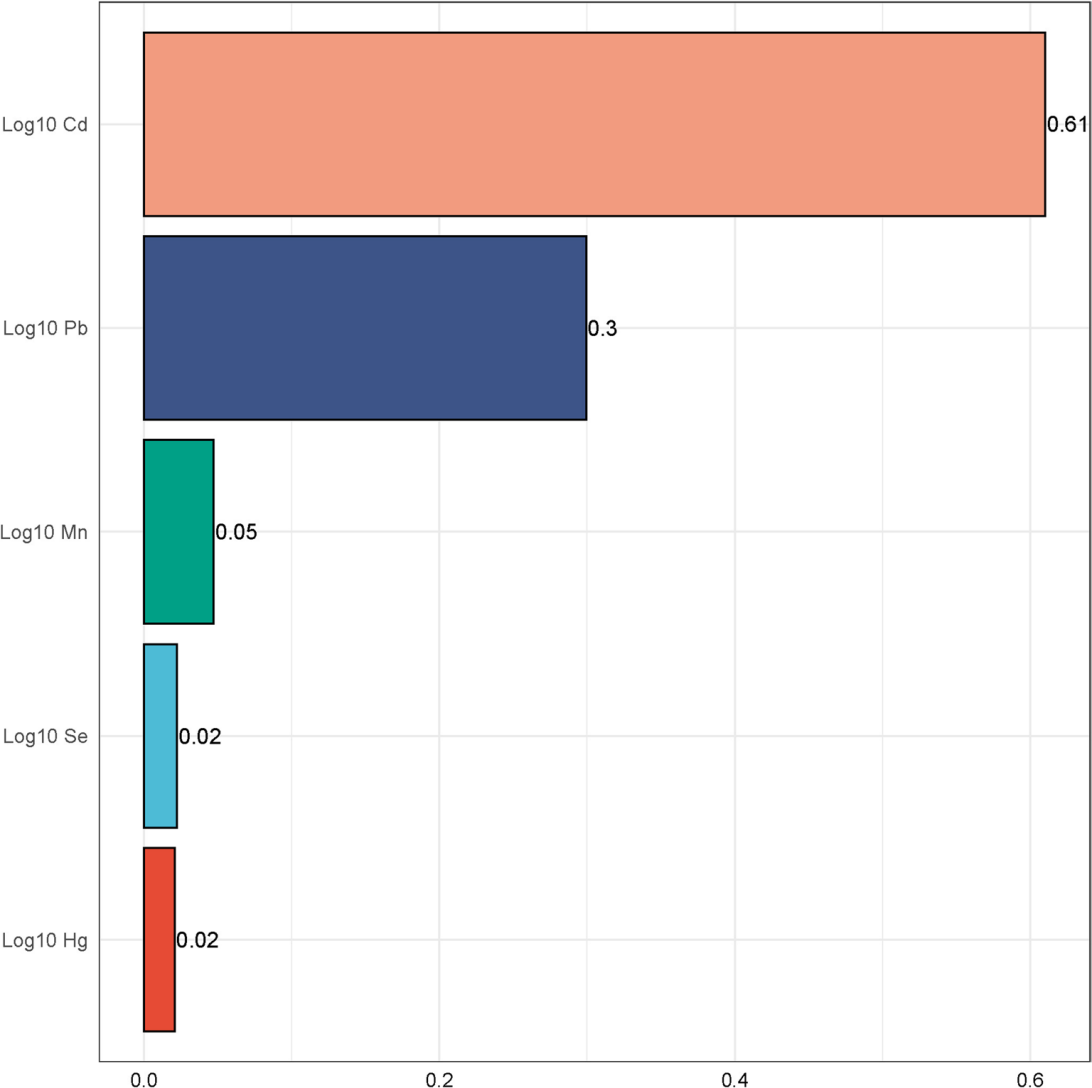
The WQS model weights of heavy metals on the odds of CHF in positive direction. This model adjusted for all covariates. *WQS* weighted quantile sum, *Mn* manganese, *Hg* mercury, *Se* selenium, *Pb* lead, *Cd* cadmium

The relationship between the comprehensive effects of five blood lead and heavy metals and CHF was investigated using the WQS model. The WQS index revealed a positive correlation between heavy metal mixtures and the odds of CHF (adjusted model: OR = 1.01, 95% CI 1.00–1.02, P = 0.032; see Supplementary Table 2). As depicted in Fig. 3, the elemental weights for Cd, Pb, Mn, Se, and Hg are 0.61, 0.30, 0.05, 0.02, and 0.02, respectively. Notably, Cd exhibits the highest weight among these elements. After adjusting for all covariates, the relative contributions of the aforementioned heavy metals remained unchanged. However, despite the low weight of selenium in the heavy metal mixture, its biological protective mechanism is substantial, and the harmful degree of Se on the WQS index is significantly less than that of Cd alone on CHF.

### Heavy metals exposure and CHF odds in the BKMR model

**Fig. 4:**
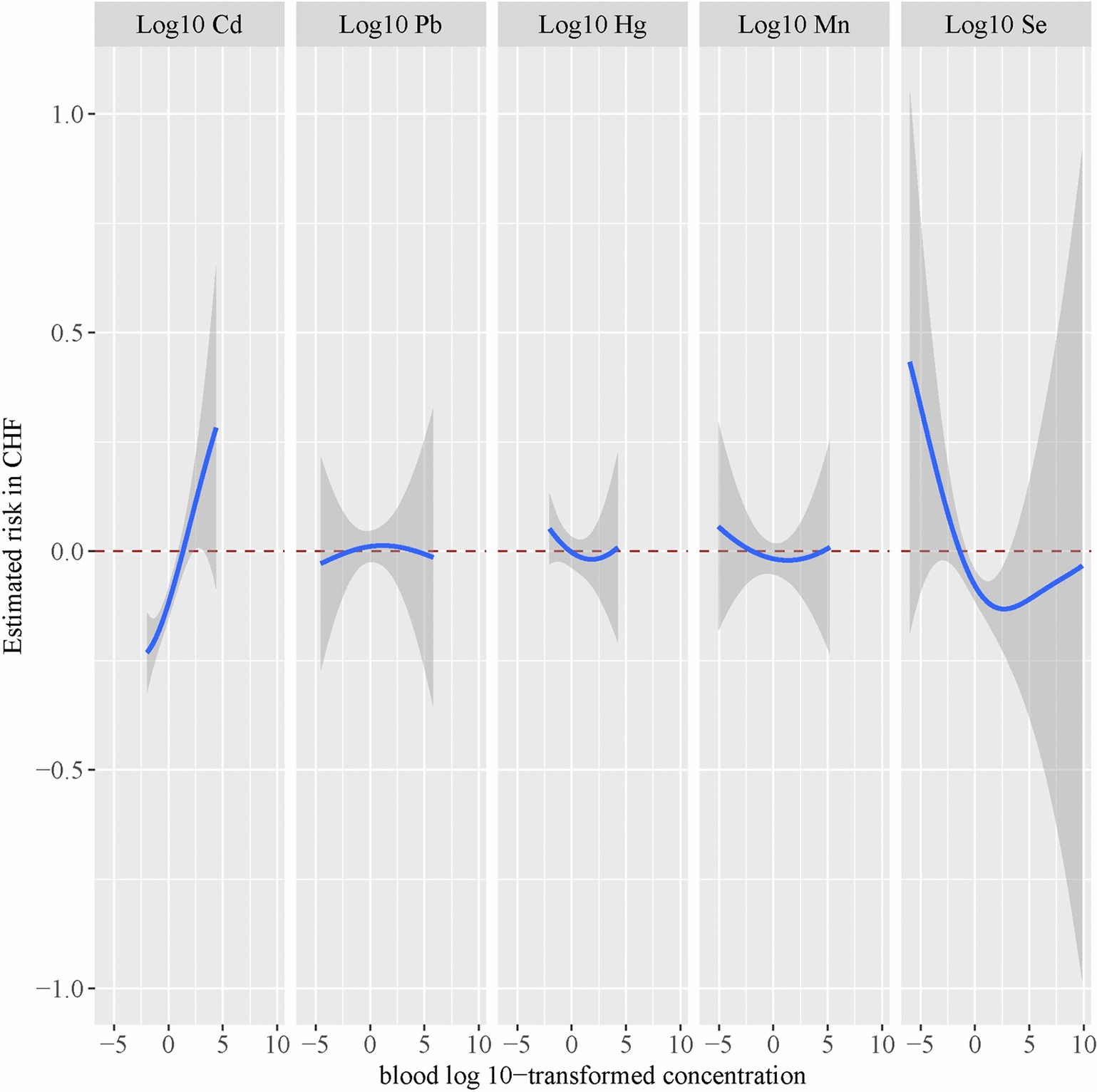
Univariate exposure–response function between each heavy metal and the odds of CHF when the other heavy metals was fixed at 50th percentiles. The model adjusted for all covariates. *Pb* lead, *Cd* cadmium, *Hg* mercury, *Se* selenium, *Mn* manganese

**Fig. 5:**
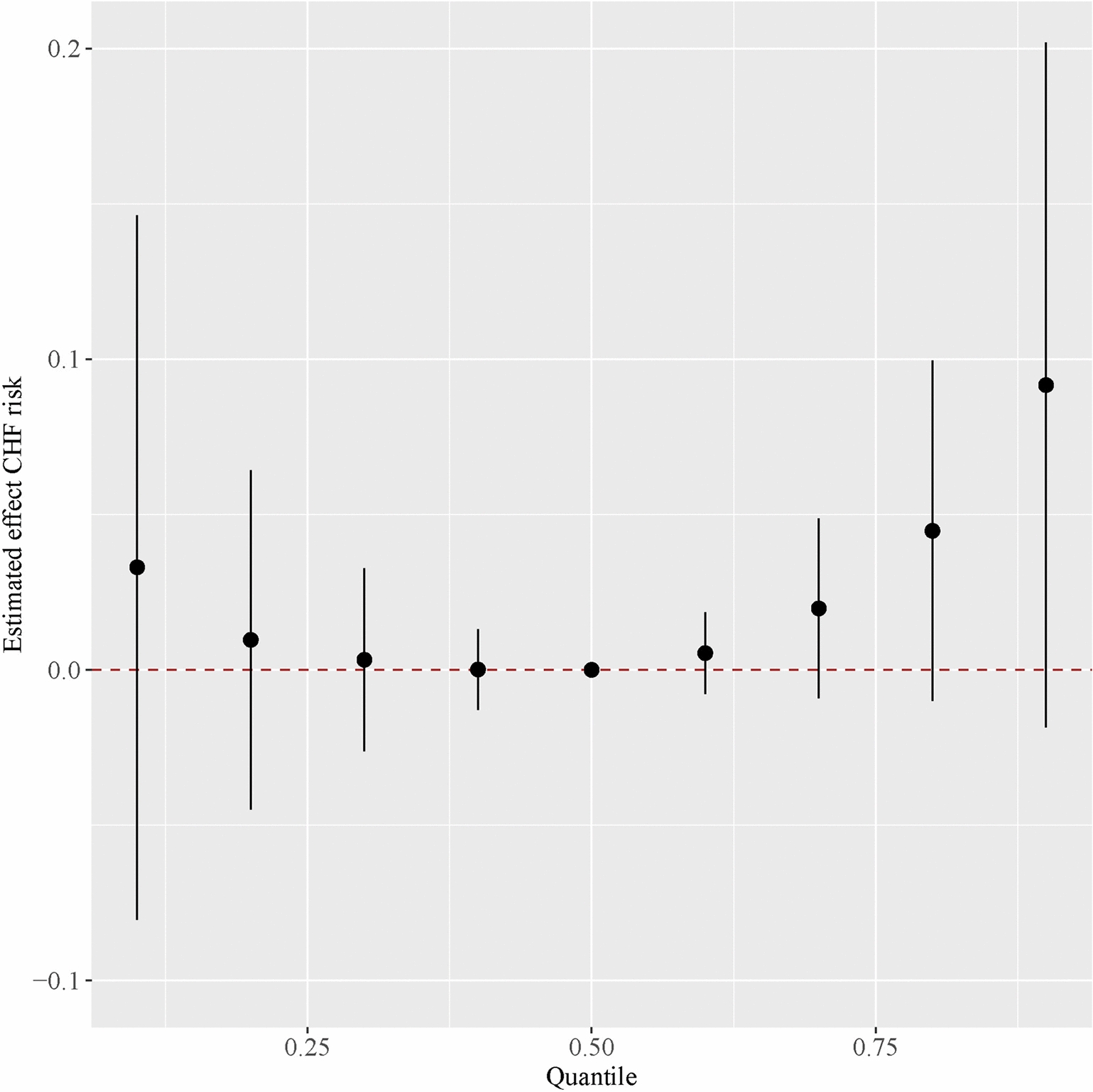
Overall effect of heavy metals mixtures on the odds of CHF in BKMR model. This plot showed the estimated difference in CHF odds and 95% confidence interval when all metals concentrations were held at particular percentiles compared to their medians. The model adjusted for all covariates. *BKMR* Bayesian kernel machine regression, *Pb* lead, *Cd* cadmium, *Hg* mercury, *Se* selenium, *Mn*, manganese

In the dose–response relationship depicted in Fig. 4 between the monometallic model and CHF, we observed a significant positive trend between blood Cd levels and CHF, while the incidence of CHF exhibited a downward trend with increasing blood Se levels. Figure 5 illustrates the metal mixture model, which shows a U-shaped correlation trend between the metal mixture and CHF when the mixture level is set at 50% as the reference value. However, since the error bars all cross over 0, this indicates no significant correlation between the metal mixture and the risk of CHF. The summary of the PIPs in the BKMR model is presented in Table 3. Among the heavy metals, serum Cd and Se concentrations have the strongest association with CHF, with PIP values of 0.72 and 0.56, respectively. Therefore, we conclude that the opposing effects of blood Cd and Se on CHF are the most pronounced within the mixture, with Cd exhibiting a harmful effect and Se a protective one. Consequently, the overall harmfulness of the metal mixture on CHF is significantly mitigated.

**Table 3:** PIPs of each heavy metal for the odds of CHF in BKMR model

heavy metal	PIP
Log10 Cd	0.72
Log10 Pb	0.13
Log10 Hg	0.19
Log10 Mn	0.14
Log10 Se	0.56

This model adjusted for all covariates

*PIP* posterior inclusion probability, *BKMR* Bayesian kernel machine regression, *Pb* lead, *Cd* cadmium, *Hg* mercury; *Se* selenium, *Mn* manganese

## Discussion

This large-scale, cross-sectional study, utilizing a nationally representative population, demonstrates a significant association between elevated serum Cd levels and the odds of CHF, while increased serum Se levels are found to be protective against CHF. However, no consistent linear correlation was observed between the remaining metals—Pb, Hg, and Mn—and CHF.

Both the BKMR and WQS models are designed to simulate the complex mixtures of metal elements found in the natural environment, thereby reflecting the impact of human exposure to heavy metal pollutants on cardiovascular diseases as they occur in the real world. However, our results indicate that the odds of CHF in patients with glucose metabolism disorders is increased by mixed pollutants, but the harm is not as pronounced as that observed with single heavy metal exposures. This suggests that different metal elements have varying susceptibilities to disease and, secondly, that not all metal elements are detrimental to health. Consequently, when considering different metal elements as part of a mixture, the original adverse effects may be significantly mitigated. These findings emphasize the need to trace the sources of metal mixtures and identify key indicators that significantly impact disease outcomes.

Previous studies have predominantly focused on reporting the association between individual blood Cd levels and blood Se levels in relation to CHF. Peter et al. [36]. Conducted a cross-sectional analysis using NHANES population data from 1999 to 2006, which included 12,049 individuals aged 30 years in the general population. The objective of this study was to assess the relationship between blood cadmium levels and the risks of stroke and heart failure. The findings revealed that, after accounting for confounding factors, elevated cadmium exposure was significantly correlated with an increased risk of both stroke and heart failure. In an observational cohort study conducted in Sweden, the association between blood cadmium levels and the incidence of heart failure and atrial fibrillation was investigated. The study enrolled 4378 participants aged 46–67 years with no prior history of heart failure or atrial fibrillation. After a follow-up period of 17 years, 143 participants developed heart failure. Multivariate Cox proportional regression analysis revealed that individuals in the Q4 group had a significantly higher risk of developing heart failure compared to those in the blood CdQ1 group, with a hazard ratio (HR) of 2.64 (95% confidence interval [CI]: 1.60–4.36) [37]. Zhang et al. conducted a cross-sectional study involving 16,311 individuals aged 20 years and above to assess the association between blood selenium levels and heart failure. The findings revealed that for every 10 ug/L increase in blood selenium level, there was a statistically significant average decrease of 5% (95% CI: 0.1%, 9.8%) in the risk of heart failure [23]. A higher blood Se level may confer protection against heart failure, conversely, a lower blood selenium level is often indicative of heart failure. The typical range for normal blood Se content is between 90 and 125 μg/L [38, 39]. However, a substantial cohort of European patients with chronic heart failure (N = 2328) demonstrated that approximately 70% of HF patients had serum Se levels below 100μ g/L, these patients exhibit diminished quality of life, impaired exercise capacity, and unfavorable prognosis [40, 41].

The divalent cation Cd replaces zinc in numerous enzymes and metalloproteins, leading to its accumulation in the liver, kidney, and other soft tissues. Consequently, these enzymes and proteins become dysfunctional, resulting in vascular endothelial dysfunction [42], chronic inflammation [43], hypertension [44], oxidative stress [45], lipid metabolism disorder [46], myocardial electrical interference [47], and cardiotoxic effects [48]. The aforementioned factors, including chronic inflammation, vascular endothelial cell injury, and oxidative stress, collectively represent the underlying mechanisms contributing to the pathogenesis of HF. As an indispensable trace element, Se plays a pivotal role in maintaining human health [49].Se serves as a crucial antioxidant defense mechanism in the human body, while its associated thioredoxin reductase system actively participates in the regeneration process of vitamin C [50, 51]. Addressing endothelial dysfunction can significantly enhance exercise capacity, considering that reduced exercise ability is a prominent symptom of heart failure [52]. The selenium-dependent system collaborates with vitamin C to counteract oxygen free radicals and safeguard endothelial function, thereby potentially preserving exercise capacity [52–55].

Our research possesses several notable advantages. Firstly, in this pioneering study, we assessed the association between a mixture of heavy metals and HF in patients with diabetes and prediabetes, a high-risk group for HF. Heavy metals, known to accumulate in the liver, kidney, and pancreas, can damage the structure and function of islet β cells at elevated concentrations, thereby exacerbating diabetes and increasing the risk of HF [56]. Our research not only evaluates the relationship between individual heavy metals and HF in individuals with abnormal glucose metabolism but also simulates the overall impact of heavy metal exposure on health outcomes in real-world settings. Secondly, we have employed diverse statistical methodologies and meticulously adjusted for potential confounding variables to enhance both the reliability and robustness of our research findings. Lastly, all data utilized in this study originates from a large-scale population-based database that adheres strictly to stringent quality control measures. However, our study also possesses certain limitations. Firstly, due to the cross-sectional design employed in this study, it is challenging to establish a definitive causal relationship between the observed variables. Secondly, we obtained only a single measurement of the blood metal index, whereas multiple measurements could enhance the precision of the estimation results. Lastly, given that our study exclusively focuses on American adults, generalizing these findings to other populations remains uncertain.

## Conclusions

This cross-sectional study demonstrates that increased Cd levels are associated with a higher odds of CHF in patients with diabetes and pre-diabetes, whereas elevated blood Se levels significantly mitigate this odds. Consequently, when patients with dysregulated glucose metabolism are exposed to a mixture of heavy metals, the likelihood of CHF, although statistically significant, is minimal and may hold limited clinical significance. The findings underscore the importance of considering the impact of individual blood metal elements on cardiac function in patients with glucose metabolism disorders.

## Supplementary Information

Supplementary material 1.

## Data Availability

Publicly available datasets were analyzed in this study. This data can be found here: https://www.cdc.gov/nchs/nhanes/index.htm.
