# Supplementary material for: Cadmium and selenium blood levels in association with congestive heart failure in diabetic and prediabetic patients: a cross-sectional study from the national health and nutrition examination survey
Source: Diabetol Metab Syndr. 2025 Jan 9;17:12. doi: 10.1186/s13098-024-01556-w (PMC11715992; doi:10.1186/s13098-024-01556-w)
Supplement: Supplementary file 1 — Supplementary material 1. [file 13098_2024_1556_MOESM1_ESM.pdf]

Supplementary table 1 Multivariate logistic regression analysis of Hg for the odds of CHF.

Exposure	Adjust Model*	
	OR (95%CI)	P-value
Log10 Hg (Per SD increment)	0.88 (0.77, 1.01)	0.064
Q1(Hg<0.41)	Reference	
Q2 (0.41<Hg to<0.78)	0.94 (0.70,1.27)	0.686
Q3 (0.78<Hg<1.63)	0.89 (0.64, 1.23)	0.472
Q4 (Hg≥1.63)	0.75 (0.52, 1.07)	0.110
Log10 Hg (Per SD increment)	0.116	

*Adjust model adjust for: gender, age, race, PIR, BMI, TC, HDL-c, eGFR, smoking status, hypertension, hyperlipidemia, lipoprotein-lowering drugs, antihypertensive drugs, Se.

Supplementary Table 2 The joint effect of heavy metals mixtures on the prevalence of CHF in WQS model.

CHF	OR	95% CI	*P*-value
Adjusted model	1.01	1.00-1.02	0.032

Adjust model adjust for: gender, age, race, PIR, BMI, TC, HDL-c, eGFR, smoking status, hypertension, hyperlipidemia, lipoprotein-lowering drugs, antihypertensive drugs.
